# H-score and ERBB2 mRNA refine identification of HER2-low and HER2-ultralow breast cancer

**DOI:** 10.1186/s13058-026-02334-4

**Published:** 2026-07-08

**Authors:** Guilherme Parisotto Sartori, Susana Ramalho, Leonardo Roberto da Silva, Guilherme Defante Telles, Facundo Zaffaroni, Geisilene Russano de Paiva, Mahira Lopes da Rosa, Tomás Reinert, Guilherme Coelho, Jovana Mandelli, Grazielle Morais Tavares, Higor Kassouf Mantovani, Vivian Vasconcelos, Ana Elisa Ribeiro da Silva Cabello, César Cabello, Carlos Barrios, Marcia Silveira Graudenz

**Affiliations:** 1 Post-Graduation Program of Medical Sciences, Federal University of Rio Grande do Sul (UFRGS), Porto Alegre, Brazil; 2 Instituto do Câncer do Hospital Tacchini, Bento Gonçalves, Brazil; 3 Grupo Brasileiro de Estudo em Câncer de Mama (GBECAM), São Paulo, Brazil; 4 Department of Obstetrics and Gynecology, Faculty of Medical Sciences, State University of Campinas (UNICAMP), Campinas, Brazil; 5 Laboratory of Aging Biology (LaBE), Biology Institute, State University of Campinas (UNICAMP), Campinas, Brazil; 6 Centers of Studies in Exercise Oncology (CEEO), State University of Campinas (UNICAMP), Campinas, Brazil; 7 Estatística Sem Segredo, Porto Alegre, Brazil; 8 Laboratory of Molecular and Investigative Pathology (LAPE), Faculty of Medical Sciences, State University of Campinas (UNICAMP), Campinas, Brazil; 9 Latin American Cooperative Oncology Group (LACOG), Porto Alegre, Brazil; 10 Grupo Diagnose Patologia e Biologia Molecular, Caxias do Sul, Rio Grande do Sul Brazil; 11 Department of Pathology, Faculty of Medical Sciences, Federal University of Rio Grande do Sul (UFRGS), Porto Alegre, Brazil

**Keywords:** Breast cancer, HER2-low, HER2-ultralow, ERBB2 mRNA expression

## Abstract

**Background:**

Accurate HER2 assessment is essential for breast cancer (BC) treatment, as it directs targeted therapy decisions and predicts patient prognosis. Even though immunohistochemistry (IHC) is widely used, manual scoring is susceptible to variability. This study was conducted to evaluate HER2 expression in HER2-negative breast cancer patients treated with neoadjuvant chemotherapy (NAC), using H-score and *ERBB2* mRNA quantification.

**Methods:**

We conducted a retrospective cohort study in breast cancer patients treated with NAC between 2017 and 2023. H-score was evaluated in 506 patients. For gene expression analyses, only tumors with available H-score and Ct ≤ 35 for all candidate reference genes were included, resulting in 173 evaluable cases for ERBB2 mRNA quantification. HER2-zero tumors were further stratified into HER2-null (H-score = 0) and HER2-ultralow (H-score 1–120) groups.

**Results:**

Among all 506 HER2-negative tumors, 54.6% were classified as HER2-low and 45.4% as HER2-zero. Within the HER2-zero subgroup, 47.4% showed measurable HER2 expression (HER2-ultralow). ERBB2 mRNA levels were significantly higher in HER2-low tumors compared to HER2-zero. Among HER2-zero, HER2-ultralow tumors had higher ERBB2 expression than HER2-null. Higher H-scores were associated with increased *ERBB2* transcript expression, according to Spearman correlation coefficient (*p* < 0.05). HER2-null tumors were more frequently triple-negative.

**Conclusions:**

Quantitative assessment by H-score and ERBB2 transcript analysis reveals heterogeneity within HER2-zero tumors, identifying a HER2-ultralow population with potentially distinct clinical behavior. These findings support the integration of continuous HER2 expression metrics into clinical decision-making and trial design, particularly in the era of HER2-targeted antibody–drug conjugates.

**Supplementary Information:**

The online version contains supplementary material available at https://doi.org/10.1186/s13058-026-02334-4.

## Background

Breast cancer represents a significant global health challenge as the leading cause of cancer mortality among women. Over the past decades, human epidermal growth factor receptor 2 (HER2) status has continually evolved and remains one of the most relevant biomarkers in breast oncology [1]. Breast cancer lacking HER2 overexpression used to be conventionally managed without HER2-directed therapy [2]. However, the development of next-generation antibody-drug conjugates (ADCs) has redefined the clinical significance of lower levels of HER2 expression [2, 3]. Trastuzumab Deruxtecan (T-DXd) has emerged as the first targeted agent to improve survival in metastatic breast cancer (BC) with low HER2 expression (IHC 1 + or 2 + without HER2 amplification by in situ hybridization), shifting the paradigm from a binary classification of HER2 status to a more nuanced spectrum [4, 5]. 

IHC has been considered the primary method for HER2 evaluation since trastuzumab approval [6]. However, its accuracy is limited by the wide dynamic range of HER2 protein expression, various pre-analytical issues and the subjective nature of IHC scoring. Recently, DESTINY-Breast06 demonstrated the efficacy of T-DXd in patients with HER2-ultralow expression (incomplete and faint membrane staining in > 0 but ≤ 10% of tumor cells), granting FDA approval in this subset of patients traditionally included in the HER2-zero group. Whether these responses reflect bystander effects of T-DXd, reading inaccuracies or tumor heterogeneity remains unclear. These findings have highlighted the existing knowledge gap in establishing a more precise threshold of HER2 expression to guide treatment decisions [7–9]. 

These limitations have prompted the development of more precise and reliable methods for guiding treatment decisions in patients with BC. In this context, the H-score, a semi-quantitative method that integrates staining intensity and the proportion of positive cells, offers a more granular assessment of HER2 expression and may improve the discrimination of biologically distinct subgroups within HER2-negative tumors. Despite its previous use in research and some clinical trials, the H-score capacity to refine HER2 classification and predict molecular features or treatment response remains underexplored in routine clinical practice [10–13]. Furthermore, molecular techniques like mRNA expression by RT-qPCR seem to be a promising approach to accurately characterize HER2-low cancer, based on its broader dynamic scope. Xu et al. demonstrated extensive ranges for *ERBB2* mRNA expression exceeding the dynamic range of conventional IHC. *ERBB2* mRNA levels alone have been recently found to be associated with pathologic response in both early-stage and metastatic settings. Patients with high levels of *ERBB2* mRNA tend to experience a better response to HER2-targeted therapies compared to those with low levels [14, 15]. 

In this study, we aimed to evaluate HER2 expression in a cohort of patients with HER2-negative breast cancer who underwent neoadjuvant chemotherapy, using H-score and quantitative analysis of ERBB2 mRNA levels. By integrating multiple assessment modalities, our goal was to refine the characterization of the HER2-low expression continuum within the HER2-negative population, particularly in differentiating between HER2-low and HER2-zero, as well as between HER2-ultralow and HER2-null breast cancer.

## Methods

### Data source

This was a retrospective cohort study including patients treated at a single tertiary hospital in Brazil (Women’s Hospital Prof. Dr. José Aristodemo Pinotti – Campinas/SP). Institutional Review Board approval of the study protocol was obtained before the study commenced (CAAE 57159722.3.2002.5404), and all patients provided written informed consent. We prospectively collected data from patient charts, including demographics, clinicopathological variables, type and duration of treatment, breast and axillary surgery, and pathological specimen analyses.

### Study population

Eligible patients had histologically confirmed stage I-III HER2-negative invasive breast carcinoma submitted to anthracycline and taxane-based neoadjuvant chemotherapy between January 2017 and December 2023, followed by surgical treatment. We excluded patients who refused or were unfit for surgery, and those lost to follow-up.

### Outcomes and measures

A standardized IHC panel (ER, PR, and HER2) was performed on pretreatment core biopsies for all patients at the time of diagnosis as part of routine clinical practice. Hormone receptors (ER/PR) were assessed by immunohistochemistry and interpreted according to ASCO/CAP guidelines 2010 [16] for samples from 2017 to June 2020 and according to ASCO/CAP guidelines 2020 [17] for samples from July 2020 to December 2023. HER2 expression was evaluated using the Dako 0485 antibody (Agilent, Santa Clara, CA, USA), a polyclonal anti-HER2 antibody, as a laboratory-developed protocol and interpreted according to the 2018 ASCO/CAP guideline [18], its 2022 updated version [19] and the scoring proposed in the DESTINY-Breast04 study [20]. Accordingly, HER2-low was defined as faint, incomplete staining in > 10% of tumor cells (IHC-score 1+) or weak-to-moderate, complete staining in > 10% of tumor cells (IHC-score 2+), with negative in situ hybridization. HER2-zero was defined as the complete absence of staining or faint, incomplete staining in ≤ 10% of tumor cells. In samples initially classified as HER2-zero, tumor samples with an H-score ≥ 1 were classified as HER2-ultralow, and those with an H-score of 0 were classified as HER2-null. A second dedicated breast pathologist performed a semi-quantitative evaluation of HER2 expression using the H-score on the newly prepared HER2 IHC slides. These slides were generated from archived formalin-fixed paraffin-embedded (FFPE) pretreatment core biopsies using the same Dako 0485 antibody. The pathologist was blinded to prior assessments. The H-score is a semi-quantitative method used to assess biomarker expression. Tumor cells were classified based on the membrane expression level and staining pattern of HER2: score 0: no staining observed or incomplete faint/barely perceptible membrane staining; score 1+: incomplete faint/barely perceptible membrane staining; score 2+: weak to moderate complete membrane staining; score 3+: intense complete membrane staining. The H-score was calculated using the following formula: H-score = [(0 × % of score 0 cells) + (1 × % of score 1 + tumor cells) + (2 × % of score 2 + tumor cells) + (3 × % of score 3 + tumor cells)], yielding a score ranging from 0 (no expression) to 300 (100% intense staining) [21]. This score represents a weighted sum of staining intensity across the cell population, providing a continuous variable for the quantitative evaluation of target protein expression [10, 11, 13]. 

Total RNA was extracted from formalin-fixed; FFPE tumor tissue sections mounted on microscope slides using the RecoverAll™ Total Nucleic Acid Isolation Kit for FFPE (Thermo Fisher Scientific), according to the manufacturer’s instructions. RNA was eluted in a final volume defined by the kit protocol and used directly for cDNA synthesis without prior quantification. cDNA synthesis was performed using the High-Capacity cDNA Reverse Transcription Kit (Applied Biosystems, Thermo Fisher Scientific). A total of 10 µL of RNA eluate was used in a final reaction volume of 30 µL, following the manufacturer’s recommendations for low-yield or precious samples. The resulting cDNA was quantified using the Qubit™ 4 Fluorometer (Thermo Fisher Scientific) with the Qubit™ ssDNA Assay Kit, and 1 ng of total cDNA was used per reaction for subsequent qPCR analysis. Quantitative PCR was conducted on a CFX96 Touch™ Real-Time PCR Detection System (Bio-Rad, Hercules, CA), with reactions prepared in duplicate using the TaqMan™ Fast Advanced Master Mix (catalog no. 4444557, Applied Biosystems, Thermo Fisher Scientific). TaqMan-FAM-labeled primer/probes were used for *ERBB2* (catalog no. Hs01001580_m1) and for the following candidate housekeeping genes: *ACTB* (catalog no. Hs01060665_g1), *GAPDH* (catalog no. Hs02786624_g1), *GUSB* (catalog no. Hs00939627_m1), *RPLPO* (catalog no. Hs00420895_gH), and *TFRC* (catalog no. Hs00951083_m1), with a final reaction volume of 20 µL. PCR conditions were 95 °C for 20 s (enzyme activation), followed by 40 cycles of 95 °C for 3 s and 60 °C for 30 s. Only samples with Ct values below 35 for all candidate housekeeping genes were considered valid for further analysis. The most stable housekeeping gene was determined using RefFinder, a web-based platform that integrates geNorm, NormFinder, BestKeeper, and the comparative ΔCt method to rank candidate reference genes. *RPLPO* was identified as the most stable and was used for normalization. *ERBB2* expression was calculated using the 2-ΔCt method, where ΔCt = Ct*ERBB2* – Ct*RPLPO* [22, 23]. 

### Statistical analyses

Only samples with available H-score values were included for H-score analyses. For gene expression, only samples with available H-Score and Ct values ≤ 35 for all candidate reference genes were included. Data distribution was assessed using the Shapiro-Wilk test. Associations between variables were assessed using Pearson’s chi-squared test (for categorical data) or Spearman’s rank correlation (for continuous or ordinal variables). Due to the presence of tied ranks, *p* values for Spearman’s test were computed using large-sample approximations. Differences in ERBB2 mRNA levels across IHC status groups were evaluated using the Mann-Whitney U test. Between-group differences in medians were estimated using the Hodges-Lehmann method when applicable. All two-sided *p* values < 0.05 were deemed to be statistically significant. No correction for multiplicity was made. Analyses were stratified by hormone receptor status (positive or negative) where appropriate. All statistical analyses were conducted using R Statistical Software (v4.3.3; R Core Team, 2024) and GraphPad Prism version 10.0.2 (GraphPad Software, San Diego, CA).

## Results

### Study population

Patient selection flowchart is depicted in Supplementary Fig. S1. Overall, 506 patients had adequate archival tumor samples, which were evaluated using the H-score. *ERBB2* mRNA expression was assessed in 449 patients (57 were excluded due to insufficient DNA). A total of 276 patients were excluded owing to candidate housekeeping genes cycle threshold (Ct) values > 35, therefore 173 tumor samples from HER2 negative breast cancer patients treated with NAC were included in the *ERBB2* mRNA expression analysis. Table 1 describes the baseline characteristics of the initial 506 patients. The majority presented with high-risk features: 475 (94%) had invasive ductal carcinoma (IDC); 250 (49.4%) were diagnosed with stage II disease and 239 (47.2%) with stage III; 496 (98.0%) had tumors classified as histological grade 2 or 3; and 355 (70.2%) had axillary lymph node involvement. A total of 324 patients (64.2%) had hormone receptor (HR)-positive tumors, while 182 (35.8%) were classified as triple-negative breast cancer (TNBC).

HER2 IHC performed on pretreatment core biopsies identified 230 tumors (45.4%) as HER2-zero and 276 (54.6%) as HER2-low. Within HER2-zero tumors, 130 (56.2%) were HR-positive and 100 (43.8%) were TNBC; for HER2-low tumors, 194 (70.3%) were HR-positive and 82 (29.7%) were TNBC (*p* = 0.0013). Among 230 patients with HER2-zero tumors, 121(52.6%) had an H-score of 0, and were classified as HER2-null. The remaining 109 patients (47.4%) had H-scores ranging from 1 to 120 and were categorized as HER2-ultralow. Among HER2-null tumors, 53 patients (43.8%) were HR-positive, and 68 (56.2%) were TNBC; in the HER2-ultralow tumors, 77 (70.6%) were HR-positive, and 32 (29.4%), were TNBC (*p* < 0.001). Regarding response to the neoadjuvant treatment, HER2-zero tumors demonstrated higher chemotherapy sensitivity, with 21.7% achieving a pathological complete response (pCR), while HER2-low tumors achieved pCR in 15.1% (*p* = 0.031). HER2-null and HER2-ultralow had pCR rates of 29% and 13.8%, respectively.

H-score distribution significantly differed between HER2-zero (*n* = 230, median H-score 0.00) and HER2-low tumors (*n* = 276, median H-score 40.00) (*p* < 0.0001; Fig. 1A). Similarly, among HR-positive subtype (*n* = 324), the median H-score was 8.00 in the HER2-zero and 50.50 in the HER2-low tumors (*p* < 0.0001; Fig. 1B). In the TNBC subtype (*n* = 182), the median H-score was 0.00 and 20.00 for HER2-zero and HER2-low tumors, respectively, also showing a statistically significant difference (*p* < 0.0001; Fig. 1C).

**Table 1:** Clinicopathological features of HER2-negative breast cancer stratified by HER2 expression

	All HER2-negative (*n* = 506)	HER2-zero (*n* = 230)	HER2-low (*n* = 276)	*p* value	HER2-null H-score 0 (*n* = 121)	HER2-ultralow H-score 1-120 (*n* = 109)	*p* value
*Histological type (%)*							
IDC	475 (93.9)	212 (92.1)	262 (95.6)	0.029^#^	112 (92.5)	100 (91.7)	0.973^#^
ILC	22 (4.3)	10 (4.4)	11 (4.0)		5 (4.1)	5 (5.0)	
Other	9 (1.8)	8 (3.5)	1 (0.4)		4 (3.4)	4 (3.3)	
*Histological grade (%)*							
1	10 (2.0)	7 (3.1)	3 (1.1)	0.025^#^	5 (4.2)	2 (4.9)	0.179^#^
2	278 (54.9)	113 (49.3)	165 (60.2)		53 (43.8)	60 (55.0)	
3	218 (43.1)	109 (47.6)	106 (38.7)		63 (52.0)	47 (43.1)	
*TNM staging group (%)*							
I	14 (2.8)	4 (1.8)	10 (3.7)	0.24^#^	3 (2.5)	1 (0.9)	0.436^#^
II	252 (49.8)	109 (47.6)	141 (51.4)		54 (44.6)	56 (51.4)	
III	240 (47.4)	116 (50.6)	123 (44.9)		64 (52.9)	52 (47.7)	
*Menopausal status (%)*							
Premenopausal	221 (43.7)	100 (43.7)	119 (43.4)	0.99^#^	63 (52.0)	70 (64.2)	0.044^#^
Postmenopausal	283 (55.9)	128 (55.9)	154 (56.2)		58 (48.0)	38 (34.8)	
Male breast cancer	2 (0.4)	1 (0.4)	1 (0.4)		0 (0)	1 (1.0)	
*Hormone receptor (%)*							
Positive	324 (64)	130 (56.2)	194 (70.3)	0.0013^#^	53 (43.8)	77 (70.6)	< 0.001^#^
Negative	182 (36)	100 (43.8)	82 (29.7)		68 (56.2)	32 (29.4)	
*RCB (%)*							
0	92 (18.2)	50 (21.7)	42 (15.2)	0.031^#^	35 (29.0)	15 (13.8)	0.002^#^
I	39 (7.7)	16 (7.0)	23 (8.4)		9 (7.4)	7 (6.4)	
II	212 (41.9)	101 (43.9)	111 (40.2)		51 (42.1)	50 (45.8)	
III	160 (31.6)	62 (27.0)	98 (35.5)		25 (20.7)	37 (34.0)	
missing	3 (0.6)	1 (0.4)	2 (0.7)		1 (0.8)	0 (0)	
*H-score*,* median*	20	0.00	0.40	< 0.001*	0.00	25	< 0.0001*

IC NOS, invasive carcinoma not-otherwise specified; TNM, tumor, node, metastasis; RCB, residual cancer burden

#Chi-square; *Mann-Whitney

**Fig. 1:**
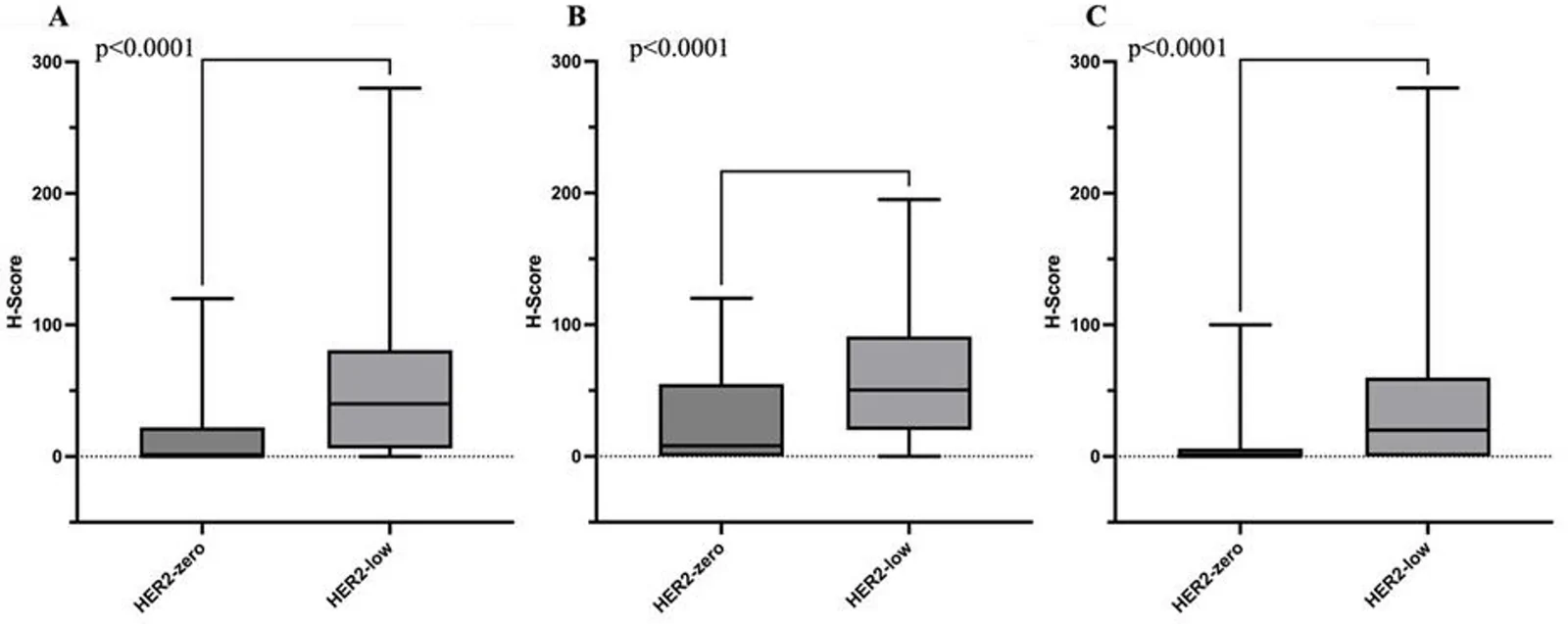
Distribution of H-score values across HER2 IHC categories, HER2-zero (*n* = 230) and HER2-low (*n* = 276). In **A** the overall study population (*n* = 506), **B** hormone receptor-positive (HR+) tumors (*n* = 324), and **C** triple-negative breast cancer (TNBC) cases (*n* = 182). The median H-score was 0.00 in the HER2-0 group and 40.00 in the HER2-low group, with a median difference of 40.00 (Hodges-Lehmann estimate: 21.00). The Shapiro-Wilk test indicated non-normal distribution in both groups (*P* < 0.0001), and the comparison using the Mann-Whitney test revealed a statistically significant difference between groups (*p* < 0.0001; U = 17,742)

### ERBB2 mRNA analysis

qRT-PCR was performed in all 506 included patients. Of these, 173 patients had samples that qualified for the ERBB2 mRNA analysis. Table 2 summarizes the clinicopathological characteristics of these 173 patients. Most patients had IDC (*n* = 164, 94.8%), presented with stage II or III disease (*n* = 169, 97.7%), and had tumors classified as histological grade 2 or 3 (*n* = 171, 98.9%). Among 173 patients, 109 (63.0%) had HR-positive tumors, and 64 (37.0%) were diagnosed with TNBC. Based on HER2 IHC analysis of pretreatment core biopsies, 77 tumors (44.5%) were classified as HER2-zero and 96 (55.5%) as HER2-low. In the HER2-zero tumors, 44 (57.1%) were HR-positive, and 33 (42.9%) were TNBC; in contrast, the HER2-low tumors included 65 (67.7%) HR-positive, and 31 (32.3%) TNBC subtype (*p* = 0.16). Within the HER2-zero tumors, 41 patients (53.2%) had an H-score of 0 and were classified as HER2-null, while the remaining 36 patients (46.8%) had H-scores ranging from 1 to 120 and were categorized as HER2-ultralow. HER2-null tumors were more frequently observed in premenopausal women (63.4% vs. 30.6% in the HER2-ultralow tumors; *p* = 0.006). Additionally, a greater proportion of HER2-null tumors were TNBC subtype (61%), whereas HER2-ultralow tumors more commonly exhibited HR positivity (77.8%; *p =* 0.001). Details of *ERBB2* and *RPL0* gene expression, including cycle threshold (Ct) and 2^− ΔCT^ values, for the overall HER2-negative population and subgroups defined by IHC and H-score are presented in Supplementary Table S1.

**Fig. 2:**
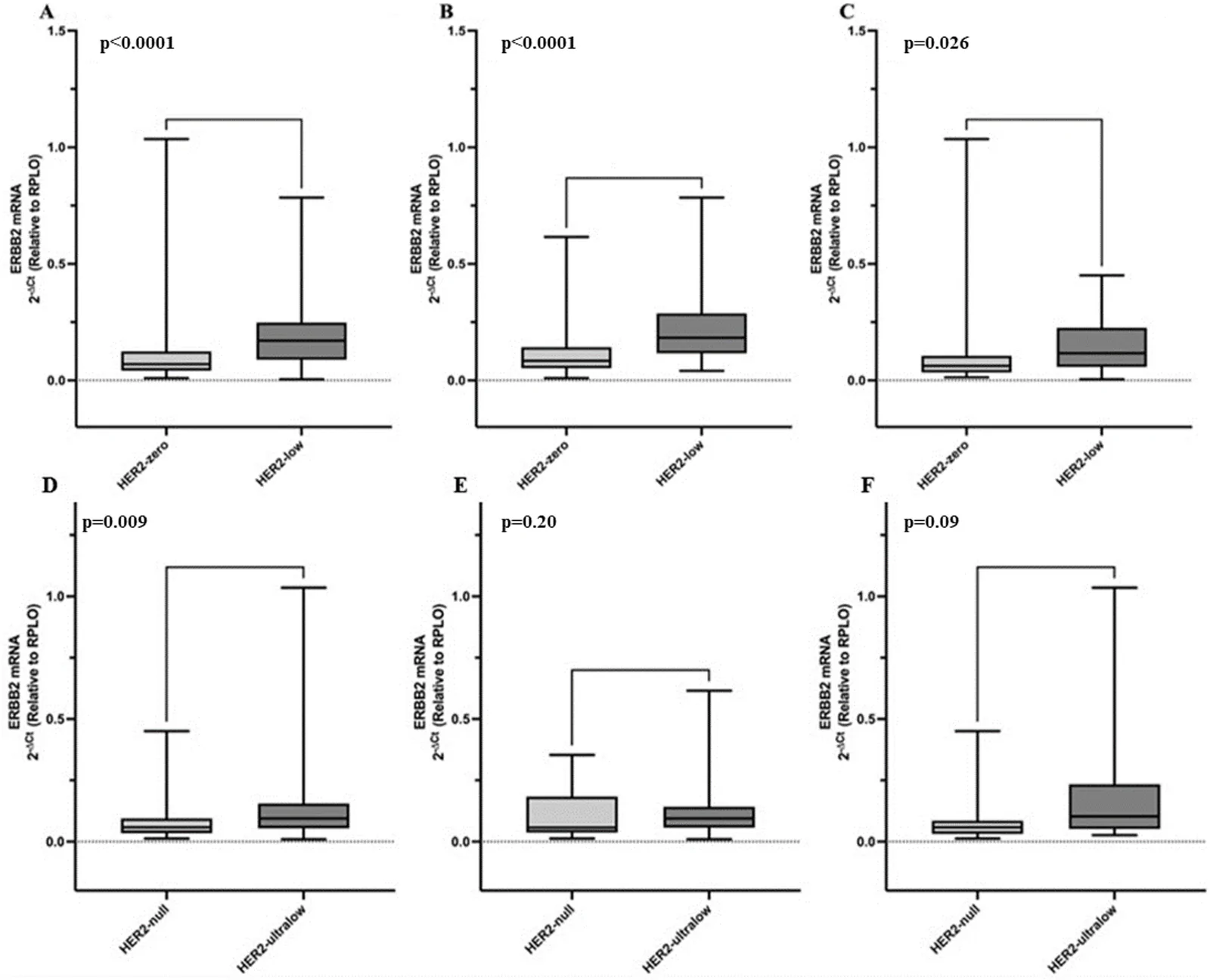
*ERBB2* mRNA expression levels according to HER2 status in the overall cohort and stratified by hormone receptor subtype. Boxplots illustrate *ERBB2* mRNA expression (2^− ΔCT^, relative to RPL0) in HER2-zero, HER2-low, HER2-null, and HER2-ultralow tumors. For HER2-zero versus HER2-low comparison, boxplot **A** illustrates the overall HER2-negative population (*n* = 173), **B** hormone receptor-positive subgroup (*n* = 106), and **C** triple-negative breast cancer subgroup (*n* = 64). For HER2-null versus HER2-ultralow comparison, boxplot **D** illustrates the overall HER2-zero population, **E** hormone receptor-positive tumors, and **F** triple-negative breast cancer (*n* = 33). *ERBB2* expression was significantly higher in HER2-ultralow tumors in the overall cohort (*p* = 0.009), with no statistically significant differences observed in the hormone receptor-positive (*p* = 0.20) or triple-negative breast cancer subgroups (*p* = 0.09) (Mann-Whitney U test). Horizontal lines indicate median values; boxes represent interquartile ranges; whiskers indicate range

Figure 2 illustrates *ERBB2* mRNA expression levels stratified by HER2 IHC status – in the overall cohort (*n* = 173), HER2-zero (*n* = 77) versus HER2-low (*n* = 96) and HER2-null (*n* = 41) vs. HER2-ultralow (*n* = 36) stratified by hormonal receptor status. *ERBB2* transcript levels were significantly lower in HER2-zero tumors across all subgroups. In the overall HER2-negative cohort (Fig. 2A) and among HR-positive subtype (Fig. 2B), *ERBB2* expression was markedly reduced in HER2-zero compared to HER2-low tumors (*p* < 0.0001 for both comparisons). A statistically significant difference was also observed. in the TNBC subtype (Fig. 2C), with lower expression in HER2-zero tumors (*p* = 0.026). In the overall cohort of HER2-zero, *ERBB2* expression was significantly lower in HER2-null tumors compared to HER2-ultralow tumors (*p* = 0.009; (Fig. 2D)). However, no statistically significant differences in *ERBB2* mRNA levels were observed in the HR-positive (Fig. 2E; *p* = 0.20) or TNBC subtypes (Fig. 2F; *p* = 0.09).

**Table 2:** Clinicopathological features of tumors with determined HER2 mRNA levels

	All HER2-negative (*n* = 173)	HER2-zero (*n* = 77)	HER2-low (*n* = 96)	*p* value	HER2-null H-score 0 (*n* = 41)	HER2-ultralow H-score 1-120 (*n* = 36)	*p* value
*Histological type (%)*							
IDC	164 (94.8)	71 (92.2)	93 (96.9)	0.55^#^	38 (92.7)	33 (91.7)	0.83^#^
ILC	6 (3.5)	3 (3.9)	3 (3.1)		2 (4.9)	1 (2.8)	
Other	3 (1.7)	3 (3.9)	0 (0)		1 (2.4)	2 (5.6)	
*Histological grade (%)*							
1	2 (1.2)	1 (1.3)	1 (1.0)	0.15^#^	1 (2.4)	0 (0)	0.05^#^
2	87 (50.3)	33 (42.9)	54 (56.3)		13 (31.7)	20 (55.6)	
3	84 (48.6)	43 (55.8)	41 (42.7)		27 (65.9)	16 (44.4)	
*TNM staging group (%)*							
I	4 (2.3)	2 (2.6)	2 (2.1)	0.86^#^	1 (2.4)	1 (2.8)	0.25^#^
II	99 (57.2)	42 (54.5)	57 (59.4)		19 (46.3)	23 (63.9)	
III	70 (40.5)	33 (42.9)	37 (38.5)		21 (51.2)	12 (33.3)	
*Menopausal status (%)*							
Premenopausal	76 (43.9)	37 (48.1)	39 (40.6)	0.35^#^	26 (63.4)	11 (30.6)	0.006^#^
Postmenopausal	97 (56.1)	40 (51.9)	57 (59.4)		15 (36.6)	25 (69.4)	
*Hormone receptor (%)*							
Positive	106 (63.0)	44 (57.1)	65 (67.7)	0.16^#^	16 (39.0)	28 (77.8)	0.001^#^
Negative	64 (37.0)	33 (42.9)	31 (32.3)		25 (61.0)	8 (22.2)	
*H-score*,* median*	20	0.00	42.50	< 0.0001*	0.00	37.50	< 0.0001*

IC NOS, invasive carcinoma not-otherwise specified; TNM, tumor, node, metastasis; RCB, residual cancer burden

#Chi-square; *Mann-Whitney

**Fig. 3:**
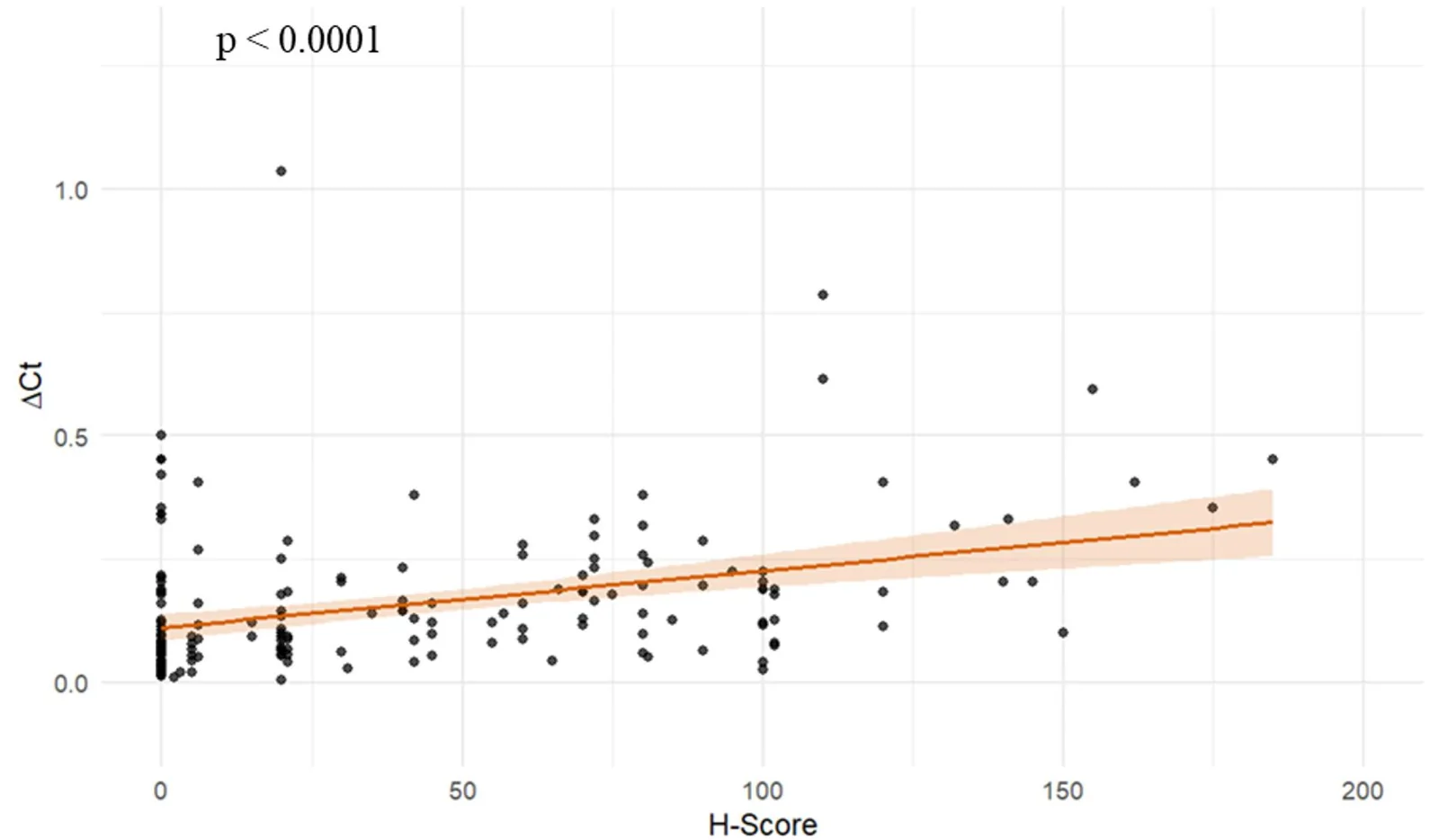
Correlation between HER2 protein expression (H-score) and *ERBB2* mRNA levels in HER2-negative breast cancer. Scatterplot illustrating the relationship between HER2 immunohistochemistry H-score and ΔCt values for *ERBB2* mRNA expression (relative to RPL0). The orange line represents the linear regression fit, with shaded area indicating the 95% confidence interval

Figure 3 shows the correlation between HER2 protein expression, measured by H-score, and *ERBB2* mRNA levels in HER2-negative breast cancer. A statistically significant positive correlation was observed according to Spearman correlation coefficient (Spearman’s ρ = 0.42; *p* value < 0.0001), indicating that higher H-scores were associated with increased *ERBB2* transcript expression.

## Discussion

In our study, we evaluated HER2 expression at the protein and transcript levels using H-score and *ERBB2* mRNA quantification in a cohort of patients with HER2-negative BC. Our results demonstrate that a significant proportion of tumors initially classified as HER2-zero exhibited measurable HER2 expression when assessed by H-score and mRNA levels. Notably, approximately half of the HER2-zero tumors displayed low but detectable protein expression (H-score 1-120), consistent with an ultralow phenotype. *ERBB2* mRNA levels were significantly higher in HER2-low compared to HER2-zero in the overall population as well as in the HR positive and TNBC subtypes. We also found a statistically significant difference in *ERBB2* mRNA levels between HER2-null and HER2-ultralow tumors. Likewise, a statistically significant correlation was observed among H-score and *ERBB2* mRNA expression. As expected, these findings corroborate the continuous biological spectrum of HER2 expression within the HER2-negative category.

### Immunohistochemistry H-score analysis

Our findings are consistent with previous reports, including the DAISY trial, in which central pathology review led to reclassification of nearly half of HER2-zero tumors as HER2-ultralow or HER2 IHC 1+ [8]. Similarly, Fernandez et al. reported central pathology review data of over 500 HER2-negative breast cancers and observed poor interobserver agreement (26% concordance in distinguishing HER2-zero from IHC 1+ [9]. These observations underscore the inadequacy of relying solely on the current IHC scoring system, designed initially to identify HER2 overexpression and amplification, in the context of emerging HER2-targeted ADCs. Notably, minimal differences in membrane staining can lead to categorical shifts with direct therapeutic implications, potentially excluding patients from treatments such as T-DXd, which has shown benefit even in tumors with ultralow HER2 expression [24]. Our data support the biological continuum of HER2 expression, which is best captured by quantitative rather than categorical scoring systems. Accordingly, a recent study by Kapil et al. proposed a continuous HER2 scoring approach that combines H-score and automated image analysis to improve patient selection for T-DXd treatment. Their findings revealed that a substantial proportion of HER2-zero tumors exhibited quantifiable HER2 expression when assessed with high-resolution digital methods and that the H-score strongly correlated with response to T-DXd, independent of standard IHC thresholds [25]. Indeed, recent advancements in AI-assisted digital pathology provide compelling support for transitioning from categorical to continuous assessment of HER2 expression.

### ERBB2 mRNA analysis

Our study provides evidence that HER2-low tumors present higher levels of ERBB2 mRNA compared to HER2-zero tumors in HER2 negative, HR positive and TNBC subtypes and our results of ERBB2 mRNA levels clearly distinguish between HER2-null and HER2-ultralow tumors. Our data correlates with those from Tyburski et al., who showed in a large cohort of 559 ER-positive, HER2-negative BC a significant difference in median HER2 mRNA values between HER2-zero and HER2-low [26]. Also, Lee et al. retrospectively collected OncotypeDx data from 1524 h positive, HER2-negative patients. They observed that the median HER2 mRNA score value of the HER2-low tumors was significantly higher than that of the HER2-zero tumors [27]. In this study, the receiver operating characteristic curve showed acceptable utility in distinguishing HER2-zero from HER2-low (area under the curve = 0.76) and HER2-null and HER2-ultralow subgroups (area under the curve = 0.81), although the overlap between subgroups was also prominent.

### Correlation between H-score and *ERBB*2 mRNA levels

In our study, we demonstrated a positive correlation between H-score and *ERBB*2 mRNA levels, revealing substantial heterogeneity even within tumors classified as HER2-zero. This aligns with recent findings using an AI-derived H-score (HAI-Score), which shows a strong correlation with HER2 mRNA measured by RNAscope (Pearson *r* = 0.85, R² = 0.71), outperforming both conventional IHC and expert pathologist scoring in predicting transcript-level expression [28]. Similarly, Wen et al. developed a deep learning–based pipeline for automated H-score quantification, which proved to be highly concordant with manual scores while offering greater reproducibility and objectivity [29]. These AI-based approaches not only reinforce the biological continuum of HER2 expression but also demonstrate that nuanced HER2 quantification—through either optimized image analysis or mRNA integration—can more accurately reflect underlying tumor biology.

### Strengths and limitations

Our study presents several strengths. First, we analyzed a large and well-characterized cohort of patients uniformly treated with neoadjuvant chemotherapy, which allowed for robust clinicopathological and molecular analyses. The integration of HER2 expression evaluation using the H-score with ERBB2 mRNA levels provided a better understanding of the HER2-negative spectrum. The use of standardized pathological review and centralized IHC evaluation adds to the methodological rigor. Additionally, our study addresses a significant clinical gap by providing quantitative evidence of biological heterogeneity within the HER2-zero category, which may have implications for patient selection in trials involving HER2-targeted ADCs. Our study has some limitations, particularly due to its retrospective nature and single-institutional setting. Evaluation of ERBB2 mRNA was limited to a subset of patients, potentially introducing selection bias and reducing statistical power in subgroup analyses. Furthermore, while the H-score adds granularity to HER2 assessment, it remains a semi-quantitative method subject to interobserver variability. Additionally, Destiny-Breast04/06 employed the Ventana Pathway 4B5 antibody, a rabbit monoclonal assay, whereas our HER2 IHC utilized the Dako A0485 polyclonal antibody, differences in clone and assay platform may influence staining intensity and concordance with the H-score ranges reported here [20, 30, 31]. The lack of RNA-based spatial profiling or digital pathology tools also limits the extent to which HER2 expression heterogeneity could be assessed at the cellular level. In addition, variability in RNA extraction from formalin-fixed, paraffin-embedded (FFPE) samples and pre-analytical factors may have contributed to dispersion in ERBB2 mRNA levels, despite stringent quality control criteria. Finally, we acknowledge that a direct comparison between conventional categorical IHC scoring (including the < 10% cutoff for HER2-ultralow), H-score quantification, and ERBB2 mRNA levels was not feasible in the present study due to the lack of standardized digital image acquisition and the inherent limitations of retrospective slide reassessment. Future studies integrating these approaches in a prospective, standardized manner may refine HER2-negative spectrum clinically.

## Conclusion

Our findings reinforce the concept that HER2-negative breast cancer is not a biologically homogeneous entity. Using quantitative tools such as H-score and *ERBB2* mRNA analysis, we demonstrate that nearly half of the tumors classified as HER2-zero exhibit measurable, albeit minimal, HER2 expression and differ significantly from HER2-null tumors in terms of hormone receptor status, gene expression, and treatment response. This refined classification uncovers a HER2-ultralow subgroup with potential clinical relevance, particularly considering the expanding role of HER2-targeted ADCs.

## Supplementary Information

Below is the link to the electronic supplementary material.

Supplementary Material 1.

Supplementary Material 2.

## Data Availability

The datasets used and/or analyzed during the current study are available from the corresponding author on reasonable request.
