# Supplementary material for: H-score and ERBB2 mRNA refine identification of HER2-low and HER2-ultralow breast cancer
Source: Breast Cancer Res. 2026 Jul 8;28:166. doi: 10.1186/s13058-026-02334-4 (PMC13628811; doi:10.1186/s13058-026-02334-4)
Supplement: Supplementary file 1 [file 13058_2026_2334_MOESM1_ESM.docx]

**Supplementary Figure S1. Study flowchart depicting the selection of patients with HER2-negative breast cancer treated with neoadjuvant chemotherapy.**

HER2 negative breast cancer patients treated with neoadjuvant chemotherapy 2017-2023 with available FFPE samples,

H score and *ERBB2* mRNA analysis and

cycle threshold (Ct) values ≤ 35

n= 173

HER2 negative breast cancer patients treated with neoadjuvant therapy 2017-2023

n= 743

HER2 negative breast cancer patients treated with neoadjuvant chemotherapy 2017-2023

and available FFPE samples

n= 551

HER2 negative breast cancer patients treated with neoadjuvant chemotherapy 2017-2023

available FFPE samples and H score analysis

n= 506

HER2 negative breast cancer patients treated with neoadjuvant chemotherapy 2017-2023

available FFPE samples, H score analysis and *ERBB2* mRNA expression.

n= 449

140 treated with neoadjuvant endocrine therapy

51 cases excluded due to lack of IC, FFPE samples or surgery, and 1 metastatic disease

45 cases without H-score analysis

57 excluded due to insufficient cDNA

276 excluded due cycle threshold (Ct) values >35
