# Supplementary material for: H-score and ERBB2 mRNA refine identification of HER2-low and HER2-ultralow breast cancer
Source: Breast Cancer Res. 2026 Jul 8;28:166. doi: 10.1186/s13058-026-02334-4 (PMC13628811; doi:10.1186/s13058-026-02334-4)
Supplement: Supplementary file 2 [file 13058_2026_2334_MOESM2_ESM.docx]

**Supplementary Table S1. ERBB2 and RPL0 gene expression metrics in HER2-negative breast cancer subgroups defined by IHC and H-score**

|  | **All**  **HER2-negative**  **(n=173)** | **HER2-0**  **(n=77)** | **HER2-low (n=96)** | **p-value** | **HER2-null**  **H-score 0**  **(n=41)** | **HER2-ultralow**  **H-score 1-120**  **(n=36)** | **p-value** |
| --- | --- | --- | --- | --- | --- | --- | --- |
| **Mean *ERBB2* CT1 and CT2**  CI 95%  SD | 28.56  24.3-36.8  2.05 | 29.10  24.85-34.40  2.01 | 28.13  24.30-36.80  2.02 | 0.0008 | 28.98  24.70-34.40  2.07 | 28.37  24.30-36.80  2.05 | 0.064 |
| **Mean *RPLO* CT1 and CT2**  CI 95%  SD | 25.37  21.20-29.85  1.71 | 25.39  21.20-29.70  1.68 | 25.36  21.60-29.85  1.74 | 0.906 | 25.25  22.15-29.05  1.57 | 25.43  21.20-29.85  1.77 | 0.500 |
| **2^-ΔCT^**  CI 95%  SD | 0.160  0.0-1.04  0.150 | 0.116  0.009-1.035  0.147 | 0.191  0.005-0.784  0.136 | <0.0001 | 0.120  0.013-0.500  0.128 | 0.176  0.005-1.035  0.150 | 0.0001 |
| Abbreviations: IHC, immunohistochemistry; CT, cycle threshold | | | | | | | |
